# Impact of exposure measurement error in air pollution epidemiology: effect of error type in time-series studies

**DOI:** 10.1186/1476-069X-10-61

**Published:** 2011-06-22

**Authors:** Gretchen T Goldman, James A Mulholland, Armistead G Russell, Matthew J Strickland, Mitchel Klein, Lance A Waller, Paige E Tolbert

**Affiliations:** 1 School of Civil and Environmental Engineering, Georgia Institute of Technology, 311 Ferst Drive, Atlanta, Georgia 30332-0512, USA; 2 Department of Environmental Health and Bioinformatics, Rollins School of Public Health, Emory University, Atlanta, Georgia 30329, USA; 3 Department of Biostatistics and Bioinformatics, Rollins School of Public Health, Emory University, Atlanta, Georgia 30329, USA

## Abstract

**Background:**

Two distinctly different types of measurement error are Berkson and classical. Impacts of measurement error in epidemiologic studies of ambient air pollution are expected to depend on error type. We characterize measurement error due to instrument imprecision and spatial variability as multiplicative (i.e. additive on the log scale) and model it over a range of error types to assess impacts on risk ratio estimates both on a per measurement unit basis and on a per interquartile range (IQR) basis in a time-series study in Atlanta.

**Methods:**

Daily measures of twelve ambient air pollutants were analyzed: NO_2_, NO_x_, O_3_, SO_2_, CO, PM_10 _mass, PM_2.5 _mass, and PM_2.5 _components sulfate, nitrate, ammonium, elemental carbon and organic carbon. Semivariogram analysis was applied to assess spatial variability. Error due to this spatial variability was added to a reference pollutant time-series on the log scale using Monte Carlo simulations. Each of these time-series was exponentiated and introduced to a Poisson generalized linear model of cardiovascular disease emergency department visits.

**Results:**

Measurement error resulted in reduced statistical significance for the risk ratio estimates for all amounts (corresponding to different pollutants) and types of error. When modelled as classical-type error, risk ratios were attenuated, particularly for primary air pollutants, with average attenuation in risk ratios on a per unit of measurement basis ranging from 18% to 92% and on an IQR basis ranging from 18% to 86%. When modelled as Berkson-type error, risk ratios per unit of measurement were biased away from the null hypothesis by 2% to 31%, whereas risk ratios per IQR were attenuated (i.e. biased toward the null) by 5% to 34%. For CO modelled error amount, a range of error types were simulated and effects on risk ratio bias and significance were observed.

**Conclusions:**

For multiplicative error, both the amount and type of measurement error impact health effect estimates in air pollution epidemiology. By modelling instrument imprecision and spatial variability as different error types, we estimate direction and magnitude of the effects of error over a range of error types.

## Background

The issue of measurement error is unavoidable in epidemiologic studies of air pollution [1]. Although methods for dealing with this measurement error have been proposed [2,3] and applied to air pollution epidemiology specifically [4,5], the issue remains a central concern in the field [6]. Because large-scale time-series studies often use single central monitoring sites to characterize community exposure to ambient concentrations [7], uncertainties arise regarding the extent to which these monitors are representative of exposure. Zeger et al. [8] identify three components of measurement error: (1) the difference between individual exposures and average personal exposure, (2) the difference between average personal exposure and ambient levels, and (3) the difference between measured and true ambient concentrations. While the former two components of error can have a sizeable impact on epidemiologic findings that address etiologic questions of health effects and personal exposure, it is the third component that is particularly relevant in time-series studies that address questions of the health benefits of ambient regulation [9].

Prior studies have suggested that the impact of measurement error on time-series health studies differs depending upon the type of error introduced [8,10,11]. Two distinctly different types of error have been identified. One type is classical error, in which measurements, *Z_t_*, vary randomly about true concentrations, ; this can be considered the case for instrument error associated with ambient monitors. That is, instrument error is independent of the true ambient level, such that . Moreover, the variation in the measurements, *Z_t_*, is expected to be greater than the variation in the true values, . Therefore, classical error is expected to attenuate the effect estimate in time-series epidemiologic studies. In contrast, under a Berkson error framework, the true ambient, , varies randomly about the measurement, *Z_t_*. This might be the case, for example, of a measured population average over the study area with true individual ambient levels varying randomly about this population average measurement. In this case, measurement error is independent of the measured population average ambient; that is, . Furthermore, the measurement, *Z_t_*, is less variable than the true ambient level, . A purely Berkson error is expected to yield an unbiased effect estimate, provided that the true dose-response is linear [3].

Several studies have investigated the impact of error type on regression models. The simultaneous impact of classical and Berkson errors in a parametric regression estimating radon exposure has been investigated [12] and error type has been assessed in a semiparametric Bayesian setting looking at exposure to radiation from nuclear testing [13,14]; however, no study to date has comprehensively assessed the impact of error type across multiple pollutants for instrument imprecision and spatial variability in a time-series context.

Error type depends on the relationship between the distribution of measurements and the distribution of true values. Because true relevant exposure in environmental epidemiologic studies is not known exactly, determination of error type is challenging; thus, here we examine the impact of error modelled as two distinctly different types: classical and Berkson. First, we examine monitor data to assess whether error is better modelled on a logged or unlogged basis. Typically, researchers investigating error type have added error on an unlogged basis (e.g. [8,11]); however, air pollution data are more often lognormal due to atmospheric dynamics and concentration levels that are never less than zero. It is plausible that true ambient exposures are distributed lognormally about a population average as well; therefore, measurement error may be best described as additive error on the log scale. We investigate the combined error from two sources that have been previously identified as relevant in time-series studies: (1) instrument precision error and (2) error due to spatial variability [9]. We limit our scope to ambient levels of pollutants measured in accordance with regulatory specifications, disregarding spatial microscale variability, such as near roadway concentrations, as well as temporal microscale variability, such as that associated with meteorological events on sub-hour time scales. Here, building on a previously developed model for the amount of error associated with selected ambient air pollutants [15], we quantitatively assess the effect of error type on the impacts of measurement error on epidemiologic results from an ongoing study of air pollution and emergency department visits in Atlanta.

## Methods

### Air Pollutant Data

**Figure 1:**
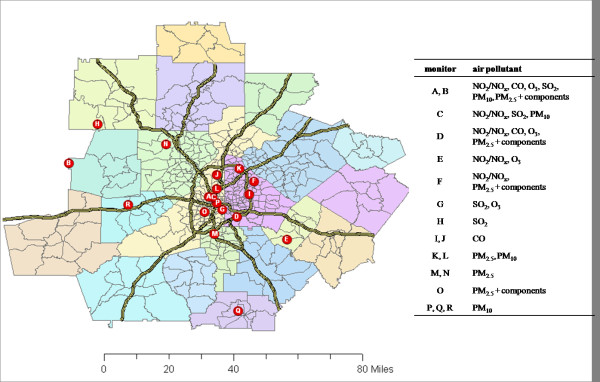
**Map of 20-county metropolitan Atlanta study area**. Census tracts, expressways, and ambient air pollutant monitoring sites are shown.

Daily metrics of 12 ambient air pollutants were studied: 1-hr maximum NO_2_, NO_x_, SO_2 _and CO, 8-hr maximum O_3_, and 24-hr average PM_10_, PM_2.5 _and PM_2.5 _components sulfate (SO_4_), nitrate (NO_3_), ammonium (NH_4_), elemental carbon (EC) and organic carbon (OC). Observations were obtained from three monitoring networks: the US EPA's Air Quality System (AQS), including State and Local Air Monitoring System and Speciation Trends Network for PM_2.5 _component measurements; the Southeastern Aerosol Research and Characterization Study (SEARCH) network [16], including the Atlanta EPA supersite at Jefferson Street [17]; and the Assessment of Spatial Aerosol Composition in Atlanta (ASACA) network [18]. Locations of the monitoring sites are shown in Figure 1.

To assess error due to instrument imprecision and spatial variability of ambient concentrations, 1999-2004 datasets were used for the 12 pollutants with data completeness for this time period (2,192 days) ranging from 82% to 97%. Data from collocated instruments were used to characterize instrument precision error. Measurement methods and data quality are discussed in detail in our prior work [15]. Distributions of all air pollutant measures more closely approximate lognormal distributions than normal distributions ([19], see Additional file 1, Table S1); therefore, additive error was characterized and modeled on a log concentration basis so that simulations with error added to a base case time-series would also have lognormal distributions.

### Measurement Error Model

The measurement error model description here highlights differences from our previous work in which error type effects were not addressed [15]. In this study, a time-series of observed data was taken to be the "true" time-series, , serving as a base case. Classical-like or Berkson-like error was added to this base case to produce a simulated time-series, *Z_t_*, that represents a population-weighted average ambient time-series. Here, the asterisk refers to a true value (i.e. without error) as opposed to a value that contains error (i.e. the simulated values in this study). The choice of which pollutant to use for the true, or base case, time-series is arbitrary, as long as an association with a health endpoint has been observed with that pollutant. To develop simulated datasets with modeled instrument and spatial error added, the following steps were taken. Base case time-series data were normalized as follows.(1)

Here,  is the normalized log concentration on day *t *and *μ*_InZ* _and *σ*_InZ* _are the mean and standard deviation, respectively, of the log concentrations over all days *t*; thus, the mean and standard deviation of  are 0 and 1, respectively. Error in  was modeled as multiplicative (i.e. additive on a log scale) as follows.(2)

Here, *ε_χt _*is the modeled error in  for day *t*, *N_t _*is a random number with distribution ~*N*(0,1) and *σ_err _*is the standard deviation of error added, a parameter derived from the population-weighted semivariance to capture the amount of error present for each pollutant, as described in the next subsection. Short-term temporal autocorrelation observed in the differences between measurements was modeled using a three-day running average of random numbers for N_t _[15].

To provide simulations of monitor data with error added (*Z_t_*), the modeled error was added to normalized data and then the normalized data with error added were denormalized in two ways: one to simulate classical-like error (i.e. classical error on a log concentration basis, referred to here as type C error) and the other to simulate Berkson-like error (i.e. Berkson error on a log concentration basis, referred to here as type B error). Simulations with type C error are generated by eq. 3.(3)

Here, *χ_t _*is the standardized simulated time-series (on the log scale) with type C error added and normal distribution . In this case of type C error, *ε_χt _*and  are independent (i.e. ). For type B error, *ε_χt _*and *χ_t _*are independent (i.e. *E*[*R(ε_χt_, χ_t_)*] = 0) and . It can be shown (see Additional file 2, eqs. S1-S6) that simulations with type B error can be generated from the true time-series by eq. 4.(4)

Here, *χ_t _*is the standardized simulated time-series (on the log scale) with type B error added and normal distribution . After the standardized simulated time-series is generated by either eq. 3 or eq. 4, the simulations are denormalized by eq. 5.(5)

For both error types, the simulated time-series (*Z_t_*) and true time-series () have the same log means (*μ*_In*Z *_= *μ*_InZ*_). For classical-like error (type C), the log standard deviation is greater for the simulated time-series than the true time-series (*σ*_In*Z *_>*σ*_InZ*_) because the simulated values are scattered about the true values. For Berkson-like error (type B), the log standard deviation is less for the simulated time-series than the true time-series (*σ*_In*Z *_<*σ*_InZ*_) because the true values are scattered about the simulated values.

### Semivariogram Analysis

To quantify the amount of error (i.e. *σ_err_*) due to instrument imprecision and spatial variability to add to the simulated time-series for each pollutant (eq. 2), we made use of the geostatistical tool of the semivariogram, which provides information on spatial autocorrelation of data and has proved useful in air pollution applications [20,21]. Here, the semivariance of the differences between normalized observations (*χ_k _*and *χ_l_*) at two locations (*k *and *l*) located a distance *h *apart is normalized by the temporal variance (variation over the time-series of observations) of the average of two normalized observations to yield a scaled semivariance, *γ*'. It can be shown that this scaled semivariance (i.e. the semivariance of normalized values) is related to the Pearson correlation coefficient (R) between normalized observations from two monitors as follows [21].(6)

Thus, *γ*' represents the spatial semivariance scaled to a quantity indicative of the range of exposures over which health risk is being assessed; it is unitless and allows for comparison across pollutants. A scaled semivariance value of 0 corresponds to perfectly correlated observations (R = 1) and a value of 1 corresponds to perfectly uncorrelated observations (R = 0).

Correlations between observations from all pairs of monitors measuring the same pollutant during 1999-2004 were calculated on a log concentration basis. Assuming the spatial variation of air pollutants to be isotropic, scaled semivariograms were constructed and modeled as a function of the distance between observations, *h*, using a sill of 1, nugget values derived from collocated measurement time-series described in previous work, and least squares regression to determine the range [15]. The estimate from the semivariogram function for each of the 660 Census tracts was weighted by the population in that tract (estimates from 2000 Census data) to derive an overall population-weighted average for each pollutant; thus, the population-weighted semivariance includes impacts of both instrument imprecision and spatial variability and represents the population-weighted average semivariance between all residences in the study area.(7)

Here,  is the population-weighted average scaled semivariance on a log scale, *p_total _*is the total population of the study area, *p_i,j _*is the sum of population in census tracts *i *and *j*, and  is the value of the semivariance function at the distance between centroids of census tracts *i *and *j*. For within-tract resident pairs, an average distance between residences was applied. Semivariograms for each of the twelve pollutants studied have been shown previously [15] and population-weighted semivariances are in Table 1. The population-weighted semivariance is related to the population-weighted correlation coefficient as follows.(8)

**Table 1:** Population-weighted scaled semivariances, , Pearson correlation coefficients, , and model parameters used in the Monte Carlo simulations to simulate amount of error (*σ_err_*) and error type (*σ*_In*Z*_/*σ*_InZ*_)

Pollutant			*σ_err_*	*σ*_In*Z*_/*σ*_InZ*_ Type B	*σ*_In*Z*_/*σ*_InZ*_ Type C
1-hr max NO_2_	0.516	0.320	1.46	0.57	1.77

1-hr max NO_x_	0.384	0.445	1.12	0.67	1.50

8-hr max O_3_	0.051	0.903	0.33	0.95	1.05

1-hr max SO_2_	0.517	0.319	1.46	0.56	1.77

1-hr max CO	0.411	0.418	1.18	0.65	1.55

24-hr PM_10_	0.192	0.678	0.69	0.82	1.21

24-hr PM_2.5_	0.100	0.819	0.47	0.90	1.11

24-hr PM_2.5_-SO_4_	0.068	0.873	0.38	0.93	1.07

24-hr PM_2.5_-NO_3_	0.140	0.754	0.57	0.87	1.15

24-hr PM_2.5_-NH_4_	0.149	0.741	0.59	0.86	1.16

24-hr PM_2.5_-EC	0.337	0.495	1.01	0.70	1.42

24-hr PM_2.5_-OC	0.175	0.702	0.65	0.84	1.19

Model parameter *σ_err _*(eq. 2) is defined to provide simulations with an amount of error such that  where  is obtained from semivariogram analysis (eqs. 6-8). The correlation between the true ambient time-series and a time-series with error added, i.e. R(ln *Z*, ln Z*), is the square root of the correlation between any two time-series, i.e. R(ln *Z_1_*, ln *Z_2_*), where each is derived by adding the same amount of error to the true ambient time-series. Since the standard deviation of *χ_t _*depends on *σ_err_*, the standard deviation of the simulated time-series relative to that of the true time-series (*σ*_In*Z*_/*σ*_InZ*_) depends on  as well. The following analytical relationships for *σ_err _*and *σ*_In*Z*_/*σ*_InZ* _were derived (see Additional file 2, eqs. S7-S10).(9)(10)

Values of *σ_err _*and *σ*_In*Z*_/*σ*_InZ* _used here can be found in Table 1.

Sets of 1000 simulated time-series with instrument and spatial error added for each pollutant for the scenarios of C and B error types were produced for the six-year period 1999-2004. In addition, simulations of CO measurement error only were generated for a range of error types with *σ*_In*Z*_/*σ*_InZ* _values between error types C and B. Scatterplots demonstrate that C and B error types defined on a log basis (i.e. In*Z *- InZ*) are independent of InZ* and In*Z*, respectively (see Additional file 3, Figure S1).

### Epidemiologic Model

Relationships between daily measures of ambient air pollution and daily counts of emergency department (ED) visits for cardiovascular disease (CVD, including ischemic heart disease, dysrhythmia, congestive heart failure, and peripheral/cerebrovascular disease) were assessed using methods described elsewhere [22] and briefly summarized here. There were 166,950 ED visits for CVD in the 20-county metropolitan Atlanta area during 1999-2004. Lag 0 associations between daily pollutant concentration and the daily count of ED visits were assessed using Poisson generalized linear models that were scaled to accounted for overdispersion. The general form of the epidemiologic model is(11)

where *Y_t _*is the count of emergency department visits, *Z_t _*is the mismeasured pollutant concentration, and *confounders_t _*is the vector of potential confounders on day *t*. The specific potential confounders included in the model were indicator variables for day-of-week, season, and when a hospital entered or left the study; cubic terms for maximum temperature and dew point; and a cubic spline with monthly knots for day of follow-up. Poisson regression yields *α *as the intercept, *β *as the log of the rate ratio associated with a unit change in pollutant concentration, and *γ *as the vector of regression coefficients for the suspected confounders included in the model. The risk ratios (*RR*) per unit of measurement change and per interquartile range (IQR) change in pollutant concentration (*Z*) are given by eq. 12 and eq. 13, respectively.(12)(13)

Using data from the central monitor, preliminary epidemiologic assessments were performed for all air pollutants and ED visits for CVD. Consistent with previous findings [22], significant positive associations were found for several traffic-related pollutants, including NO_x_, CO and EC. For the measurement error analysis described here, we used 1-hr maximum CO data as our base case, representing in our analysis a true time-series and the measured risk ratio the true association. In this way, the exposure and health outcome values that we chose to represent true time-series have distributional characteristics expected of ambient air pollution and ED visit data. Simulations with measurement error added to the base case were used to evaluate the impact of measurement error on the epidemiologic analyses. A Monte Carlo approach was used to assess uncertainty. As already described, the relationship between this base case time-series and a simulated time-series is that expected of the average relationship between the true ambient time-series for all people and a population-weighted average time-series based on measurements in terms of error amount, with different error types evaluated. A percent attenuation in risk ratio (toward the null hypothesis of 1) is calculated as follows, with RR* representing the true risk ratio (obtained from the base case Poisson regression) and *RR *representing the risk ratio obtained using simulated population-weighted time-series.(14)

## Results

### Distribution of Measurement Error Simulations

**Figure 2:**
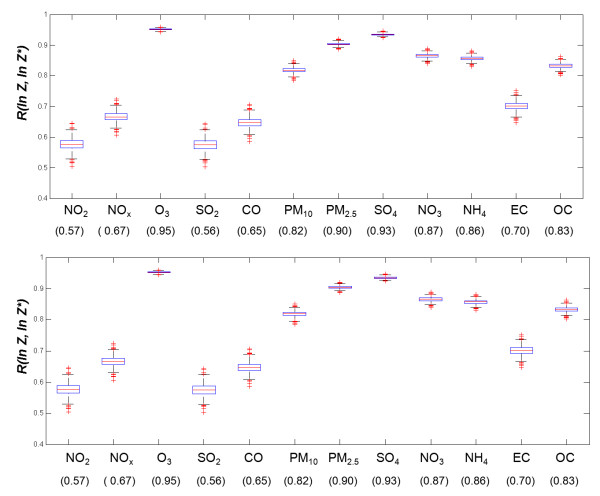
Boxplots of *R*(In*Z*, InZ*), with expected correlation coefficients shown in parentheses for 1000 simulated data time-series of error type C (top panel) and type B (bottom panel) simulations.

Analysis of the distributions of correlation coefficients between the true log concentrations (i.e. the base case) and the simulated log concentrations, R(In*Z*, InZ*), for 1000 simulations for each pollutant and each error type demonstrates that the simulations contain on average the desired amounts and types of error (Figure 2, see Additional file 4, Figure S2 for distribution of error type results). Wider distributions were observed for more spatially heterogeneous pollutants.

### Impact of Error on Health Risk Assessment

For the base case of 1-hour maximum CO measurements and CVD outcomes, a RR per ppm of 1.0139 was observed, with a 95% confidence interval (CI) of 1.0078-1.0201 and a p-value of 0.000009. With an IQR of 1.00 ppm, the RR per IQR and corresponding CI are the same as those on a per unit of measurement basis for our base case. For epidemiologic models using the time-series with simulated error added, the RR and CI results are not the same on a per measurement unit basis and a per IQR basis because the IQR of the simulated values is not 1. As expected, the simulated time-series with error type C has a greater IQR than the base case since this error is scattered about the true values, and the simulated time-series with error type B has a lower IQR than the base case since this error is scattered about the simulated values. Results of 1000 epidemiologic models for each of 12 air pollutants and two error scenarios are summarized in Table 2. The reported p-values represent those calculated from average z-score statistics and 95% confidence intervals were calculated using the asymptotic standard error estimates obtained from the regression model.

**Table 2:** Summarized epidemiologic model results with the magnitude of error representative of error associated with using a population-weighted average for each pollutant added to the base case (RR* = 1.0139, 95% CI = 1.0078-1.0201, p-value = 0.000009, IQR = 1.00 ppm)

pollutant	RR per ppm (95% CI)	IQR (ppm)	RR per IQR (95% CI)	p-value
**Error Type C simulations**				

1-hr max NO_2_	1.0011 (0.9998-1.0023)	1.84	1.0020 (0.9997-1.0042)	0.0957

1-hr max NO_x_	1.0024 (1.0003-1.0046)	1.51	1.0037 (1.0005-1.0070)	0.0251

8-hr max O_3_	1.0114 (1.0060-1.0169)	1.05	1.0120 (1.0063-1.0178)	0.00004

1-hr max SO_2_	1.0011 (0.9998-1.0023)	1.84	1.0019 (0.9997-1.0042)	0.0966

1-hr max CO	1.0021 (1.0002-1.0040)	1.57	1.0033 (1.0003-1.0063)	0.0342

24-hr PM_10_	1.0063 (1.0025-1.0102)	1.20	1.0076 (1.0030-1.0122)	0.0013

24-hr PM_2.5_	1.0094 (1.0045-1.0142)	1.10	1.0103 (1.0049-1.0156)	0.000157

24-hr PM_2.5_-SO_4_	1.0107 (1.0054-1.0159)	1.07	1.0114 (1.0058-1.0170)	0.000066

24-hr PM_2.5_-NO_3_	1.0079 (1.0035-1.0123)	1.14	1.0090 (1.0040-1.0141)	0.00040

24-hr PM_2.5_-NH_4_	1.0076 (1.0033-1.0119)	1.15	1.0088 (1.0038-1.0137)	0.00050

24-hr PM_2.5_-EC	1.0032 (1.0006-1.0057)	1.42	1.0045 (1.0009-1.0081)	0.0140

24-hr PM_2.5_-OC	1.0068 (1.0028-1.0108)	1.18	1.0080 (1.0033-1.0128)	0.00090

**Error Type B simulations**				

1-hr max NO_2_	1.0182 (1.0041-1.0325)	0.51	1.0092 (1.0021-1.0165)	0.0112

1-hr max NO_x_	1.0169 (1.0056-1.0284)	0.61	1.0103 (1.0034-1.0172)	0.0034

8-hr max O_3_	1.0142 (1.0075-1.0208)	0.94	1.0133 (1.0070-1.0195)	0.000027

1-hr max SO_2_	1.0182 (1.0041-1.0325)	0.51	1.0092 (1.0021-1.0164)	0.0114

1-hr max CO	1.0172 (1.0053-1.0292)	0.59	1.0101 (1.0031-1.0171)	0.0044

24-hr PM_10_	1.0152 (1.0068-1.0236)	0.78	1.0117 (1.0053-1.0182)	0.00030

24-hr PM_2.5_	1.0144 (1.0073-1.0217)	0.88	1.0127 (1.0064-1.0190)	0.000074

24-hr PM_2.5_-SO_4_	1.0143 (1.0074-1.0211)	0.92	1.0130 (1.0068-1.0193)	0.000039

24-hr PM_2.5_-NO_3_	1.0147 (1.0071-1.0225)	0.83	1.0122 (1.0059-1.0186)	0.000152

24-hr PM_2.5_-NH_4_	1.0148 (1.0070-1.0226)	0.82	1.0121 (1.0058-1.0185)	0.000175

24-hr PM_2.5_-EC	1.0165 (1.0060-1.0271)	0.65	1.0106 (1.0038-1.0174)	0.0021

24-hr PM_2.5_-OC	1.0150 (1.0069-1.0232)	0.79	1.0119 (1.0055-1.0183)	0.00030

**Figure 3:**
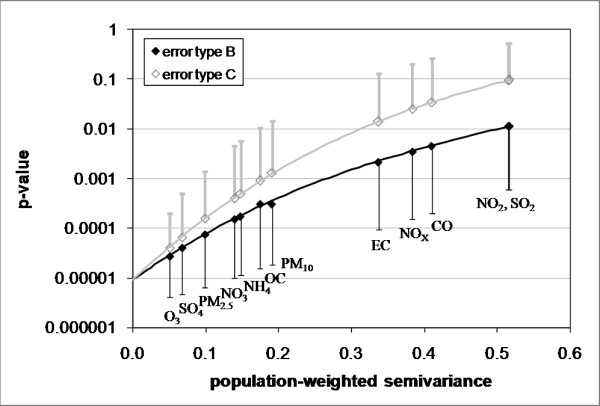
**P-values versus population-weighted semivariance**. Half-bars denote standard deviations for 1000 error simulations.

When instrument imprecision and spatial variability error were added as error type C, the average IQR of simulated time-series was greater than the IQR of the base case for all pollutants; for error type B, the average IQR of simulated time-series was less than the IQR of the base case for all pollutants. As expected, adding error to the base case resulted in a reduction of significance (i.e. a higher p-value) for both error types, as shown graphically in Figure 3. The greater the amount of error (i.e. the greater the population-weighted semivariance), the greater the reduction in significance observed. Primary pollutants (SO_2_, NO_2_/NO_x_, CO, and EC) had more error than secondary pollutants and those of mixed origin (O_3_, SO_4_, NO_3_, NH_4_, PM_2.5_, OC, and PM_10_) due to greater spatial variability. Regarding error type, there was a greater reduction of statistical significance when error type was modeled as type C than when error type was modeled as type B. For NO_2 _and SO_2_, which have the largest amount of measurement error, there was a loss of significance (p-value > 0.05) when error was modeled as error type C.

**Figure 4:**
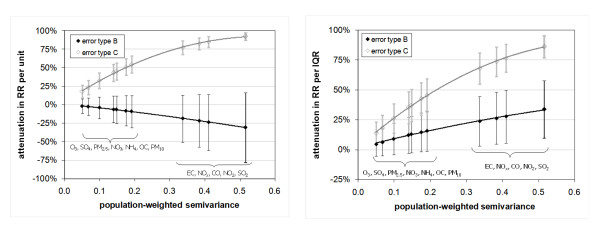
**Percent attenuation in risk ratio per ppm (left panel) and per IQR (right panel) due to error versus population-weighted semivariance**. Bars denote standard deviations for 1000 error simulations. Pollutant labels are in order of increasing population-weighted semivariance.

Risk ratio results for the two error types are plotted in Figure 4 on a percent attenuation basis. RR per unit of measurement decreased, and attenuation increased, with increasing error added (i.e. increasing population-weighted semivariance) when the error was of type C. However, RR per unit increased, with increasing bias away from the null, with increasing error added when error was of type B. For NO_2 _and SO_2_, which had the most measurement error, the attenuation was 92% when modeled as error type C and biased away from the null by 31% when modeled as error type B. On a per IQR basis, variation in the RR estimates between error types was much less dramatic. Both error types C and B led to lower RR estimates (i.e. bias towards the null). For NO_2 _and SO_2_, which again had the most measurement error, the attenuation was 86% when modeled as type C and 34% when modeled as type B error. For error type B there was a wider distribution of results than for type C error.

**Figure 5:**
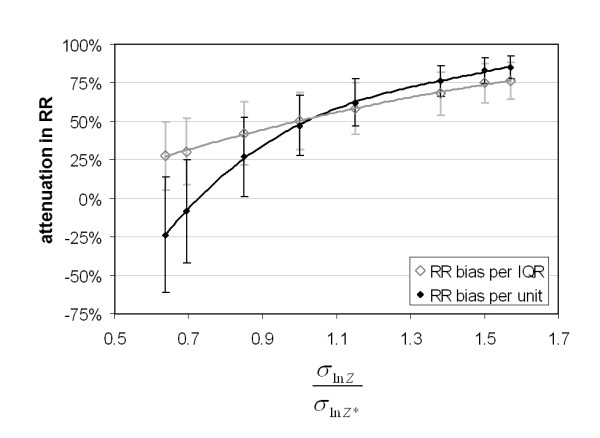
**Percent attenuation in risk ratio per unit of measurement (ppm) and per IQR for CO error simulations ( = 0.411) with incremental changes in error type ranging from type B (*σ*_In*Z*_/*σ*_InZ* _= 0.65) to type C (*σ*_In*Z*_/*σ*_InZ* _= 1.55)**. Bars denote standard deviations for 1000 simulations.

To assess a range of error types, simulations were generated with values of *σ*_In*Z*_/*σ*_InZ* _ranging from that of error type C to that of type B (eq. 10) for the case of an amount of error representative of CO ( = 0.411). Epidemiologic model results for RR attenuation are shown in Figure 5. On a per unit of measurement (ppm) basis, RR attenuation increased from -24% (i.e. a bias away from the null) for type B error to 85% for type C error. On a per IQR basis, RR attenuation increased from 28% for type B error to 85% for type C error. It is interesting to note that for *σ*_In*Z*_/*σ*_InZ* _the error (*Z *- Z*) is independent of Z (i.e. R(*Z *- Z*, *Z*) = 0) and the RR per unit attenuation is 0. This is the expected result when error is the Berkson type on an unlogged basis.

## Discussion

The results demonstrate that error type affects the reduction in significance as well as the RR estimate in the epidemiologic analysis. Moreover, the results demonstrate a profound effect of error type on the RR estimate per unit of measurement. The RR per unit of measurement estimate is increased by the presence of type B error; that is, there is a bias away from the null. To better understand these results, we estimate the attenuation in the effect estimator β (eq. 11) in the absence of confounders from the first-order linear regression coefficient (*m*) of error (Z-Z*) versus Z as follows.(15)

For RR estimates near 1 (i.e. β values near 0) as is the case in this study, the predicted attenuation in RR is approximately given as follows.(16)(17)

**Figure 6:**
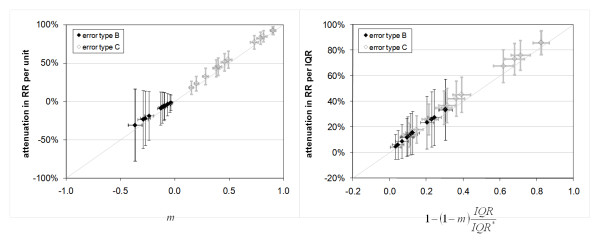
**Attenuation in the risk ratio per unit of measurement (left panel) and per IQR (right panel) due to the introduction of measurement error, modeled both as type B and type C error**. Ranges denote standard deviations for 1000 simulations. One-to-one line is also shown.

Epidemiologic model results are compared with the predictions of eq. 16 and eq. 17 for all pollutants and both error types (Figure 6). The degree to which the epidemiologic results differ from these predictions likely indicates the degree to which confounding variables are affecting results. As shown by the 1:1 line in Figure 6, there is strong agreement between the attenuation predicted by analysis of the error model results (i.e. *m *and *IQR*) and that obtained from the epidemiologic model.

In this study, in which quantification of error is based on the variability between monitors, error due to spatial variation is much greater than error due to instrument imprecision, particularly for primary air pollutants [15]. Conceptually, therefore, we speculate that this error is more likely of the Berkson type, with true values varying randomly about a population-weighted average represented by the base case. If spatial error is best described by the Berkson-like type defined on a log basis (our error type B) and the mean of the measurements is the same mean as the true values, we estimate there to be a 24% to 34% attenuation in RR per IQR estimates (Figure 4, right panel), and a 19% to 31% bias away from the null in RR estimates on a per unit of measurement basis (Figure 4, left panel), for the primary pollutants studied (SO_2_, NO_2_/NO_x_, CO, and EC) when using a population-weighted average as the exposure metric. For the secondary pollutants and pollutants of mixed origin (O_3_, SO_4_, NO_3_, NH_4_, PM_2.5_, OC, and PM_10_), we estimate a 5% to 15% attenuation in RR per IQR estimates and a 2% to 9% bias away from the null in RR estimates on a per unit of measurement basis. We are currently investigating different methods for estimating actual error type based on simulated pollutant fields trained to have all of the characteristics, including the pattern of spatial autocorrelation, expected of true pollutant fields.

This study addresses error between measured and true ambient concentrations. Our results are consistent with previous finding that suggest that Berkson error, as defined on an unlogged scale (additive), produces no bias in the effect estimate [8,11] as shown in Figure 5; however, Berkson-like error defined on a log basis (multiplicative) can lead to risk ratio estimates per unit increase that are biased away from the null (although with a reduction in significance). Thus, the direction and magnitude of the bias are functions of error type. With the multiplicative error structure used here in conjunction with a linear dose response, large "true" values of air pollution would likely be underestimated, resulting in an overestimation of pollution health effects. We have shown how multiple air pollution measurements over space can be used to quantify the amount of error and provide a strategy for evaluating impacts of different types of this error. The results suggest that estimating impacts of measurement error on health risk assessment are particularly important when comparing results across primary and secondary pollutants as the corresponding error will vary widely in both amount and type depending on the degree of spatial variability. These results are suggestive of error impacts one would have from time-series studies in which a single measure, such as the population-weighted average, is used to characterize an urban or regional population exposure. The methodology used here can be applied to other study areas to quantify this type of measurement error and quantify its impacts on health risk estimates.

## Conclusions

Health risk estimates of exposure to ambient air pollution are impacted by both the amount and the type of measurement error present, and these impacts vary substantially across pollutants. By modeling combined instrument imprecision and spatial variability over a range of error types, we are able to estimate a range of effects of these sources of measurement error, which are likely a mixture of both classical and Berkson error types. This study demonstrates the potential impact of measurement error in an air pollution epidemiology time-series study and how this impact depends on error type and amount.

## List of Abbreviations

SO4: sulfate; NO3: nitrate; NH4: ammonium; EC: elemental carbon; OC: organic carbon; AQS: US EPA's Air Quality System; SEARCH: the Southeastern Aerosol Research and Characterization Study; ASACA: Assessment of Spatial Aerosol Composition in Atlanta; ED: emergency department; CVD: cardiovascular disease; RR: risk ratio; IQR: interquartile range; CI: confidence interval.

## Competing interests

The authors declare that they have no competing interests.

## Authors' contributions

GG carried out measurement error simulations and data analyses. JM led the study design and oversaw all aspects of the research. AG provided guidance on air pollutant measurements and spatial analysis. MS carried out epidemiologic analyses and interpretation. MK and LW provided input on issues of epidemiologic modeling and biostatistics, respectively. PT led the collection of the health data and reviewed all findings. All authors contributed to writing and revising the manuscript and approve of the final manuscript.

## Supplementary Material

Additional file 1 **Power Transformation Analysis**. Click here for file

Additional file 2 **Derivations of equations in text for error models**. Click here for file

Additional file 3**Scatterplots of CO error ( = 0.411) versus InZ* for error type C (left panel) and versus In*Z *for error type B (right panel)**.Click here for file

Additional file 4 Boxplots of R(*ε*_In*Z*_, InZ*) for 1000 simulated data time-series of error type C (top panel) and R(*ε*_In*Z*_, In*Z*) for 1000 simulated data time-series of error type B (bottom panel). Click here for file
