# Supplementary material for: Impact of exposure measurement error in air pollution epidemiology: effect of error type in time-series studies
Source: Environ Health. 2011 Jun 22;10:61. doi: 10.1186/1476-069X-10-61 (PMC3146396; doi:10.1186/1476-069X-10-61)
Supplement: Additional file 1 — Power Transformation Analysis. [file 1476-069X-10-61-S1.PDF]

<u>Additional File 1.</u> Power Transformation Analysis.

The Hinkley d_λ statistic, defined as $\frac{|mean - median|}{IQR}$, provides information on the normality of a transformation for a given choice of λ, where $\lambda = 0$ represents a log transformation and $\lambda = 1$ represents untransformed data. The smaller d_λ, the closer the data transformation is to a normal distribution. Hinkley d_λ statistics are shown for daily measurements at the central monitoring site over the period 1999-2004.

Table S1. Hinkley d_λ statistics for λ = 0 and λ = 1.

	d_λ Measurement Data	
**Pollutant**	**λ = 0**	**λ = 1**
1-hr max NO_2	0.069	0.079
1-hr max NO_x	0.058	0.376
8-hr max O_3	0.121	0.095
1-hr max SO_2	0.050	0.271
1-hr max CO	0.080	0.388
24-hr PM_{10}	0.018	0.140
24-hr $PM_{2.5}$	0.038	0.134
24-hr $PM_{2.5}$-SO_4	0.0009	0.252
24-hr $PM_{2.5}$-NO_3	0.043	0.333
24-hr $PM_{2.5}$-NH_4	0.024	0.179
24-hr $PM_{2.5}$-EC	0.019	0.233
24-hr $PM_{2.5}$-OC	0.002	0.204

$$T_1(x) = \begin{cases} x^\lambda, & \lambda > 0 \\ \ln(x), & \lambda = 0 \end{cases}$$

where *x* is a daily measurement of pollutant concentration
