# Supplementary material for: Impact of exposure measurement error in air pollution epidemiology: effect of error type in time-series studies
Source: Environ Health. 2011 Jun 22;10:61. doi: 10.1186/1476-069X-10-61 (PMC3146396; doi:10.1186/1476-069X-10-61)
Supplement: Additional file 2 — Derivations of equations in text for error models. [file 1476-069X-10-61-S2.PDF]

Additional File 2. Derivations of equations in text for error models

Z^* = true ambient, log-normally distributed; given
Z = measured ambient, log-normally distributed; simulated

1. Type C error model

$$\chi^* = \frac{\ln Z^* - \mu_{\ln Z^*}}{\sigma_{\ln Z^*}} \qquad \sim N(0,1) \qquad (S1)$$

$$\chi = \chi^* + \varepsilon_\chi \qquad \sim N(0, \sigma_\chi = \sqrt{1+\sigma_{err}^2}\,) \qquad (S2)$$

where $\varepsilon_\chi = N\ \sigma_{err}$ $\qquad \sim N(0, \sigma_{err})$

N = random number $\qquad \sim N(0,1)$

$$\ln Z = \chi\sigma_{\ln Z^*} + \mu_{\ln Z^*} \qquad \sim N(\mu_{\ln Z^*}, \sigma_{\ln Z} = \sigma_{\ln Z^*}\sqrt{1+\sigma_{err}^2}\,) \qquad (S3)$$

2. Type B error model

$$\chi^* = \frac{\ln Z^* - \mu_{\ln Z^*}}{\sigma_{\ln Z^*}} = \chi + \varepsilon_\chi \qquad \sim N(0,1) \qquad (S4)$$

$$\chi = \frac{\chi^* + \varepsilon_\chi}{1+\sigma_{err}^2} \qquad \sim N(0, \sigma_\chi = \frac{1}{\sqrt{1+\sigma_{err}^2}}) \qquad (S5)$$

$$\ln Z = \chi\sigma_{\ln Z^*} + \mu_{\ln Z^*} \qquad \sim N(\mu_{\ln Z^*}, \sigma_{\ln Z} = \frac{\sigma_{\ln Z^*}}{\sqrt{1+\sigma_{err}^2}}) \qquad (S6)$$

3. Relationship between Pearson correlation coefficient and amount of error added (σ_{err})

$$\overline{R}(\chi, \chi^*) = \sqrt{\overline{R}(\chi_i, \chi_j)} = \frac{1}{\sqrt{1+\sigma_{err}^2}} \qquad (S7)$$

$$\sigma_{err} = \sqrt{\frac{1-\overline{R}(\chi_i, \chi_j)}{\overline{R}(\chi_i, \chi_j)}} \qquad (S8)$$

4. Population-weighted semivariance and Pearson correlation coefficient

semivariance: $$\overline{\gamma'}(\chi_i, \chi_j) = \frac{1}{p_{total}}\left(\sum_{i}^{660}\sum_{j=i+1}^{660} p_{i,j}\gamma'_{i,j}(h) + \sum_{i}^{660} p_i\gamma'_i(r)\right) \qquad (S9)$$

where $\gamma'_{i,j}$ = inter-tract semivariance, based on distance h between tract i and j centroids

γ'_i = intra-tract semivariance, estimated from average radius r of census tract i

p_{total} = total number of pairs of residences = ½ ($n_{total}^2 - n_{total}$)

$p_{i,j}$ = ½ (n_i x n_j) ; p_i = ½ ($n_i^2 - n_i$)

n_i = population of tract i; n_{total} = total population

Pearson correlation coefficient: $$\overline{R}(\chi_i, \chi_j) = \frac{1-\overline{\gamma'}(\chi_i, \chi_j)}{1+\overline{\gamma'}(\chi_i, \chi_j)} \qquad (S10)$$
