# Supplementary material for: Impact of exposure measurement error in air pollution epidemiology: effect of error type in time-series studies
Source: Environ Health. 2011 Jun 22;10:61. doi: 10.1186/1476-069X-10-61 (PMC3146396; doi:10.1186/1476-069X-10-61)

Additional File 3. Figure S1. Scatterplots of CO error ($\overline{\gamma'} = 0.411$) versus $\ln Z^*$ for error type C (left panel) and versus $\ln Z$ for error type B (right panel). Black lines indicate lines of linear regression.

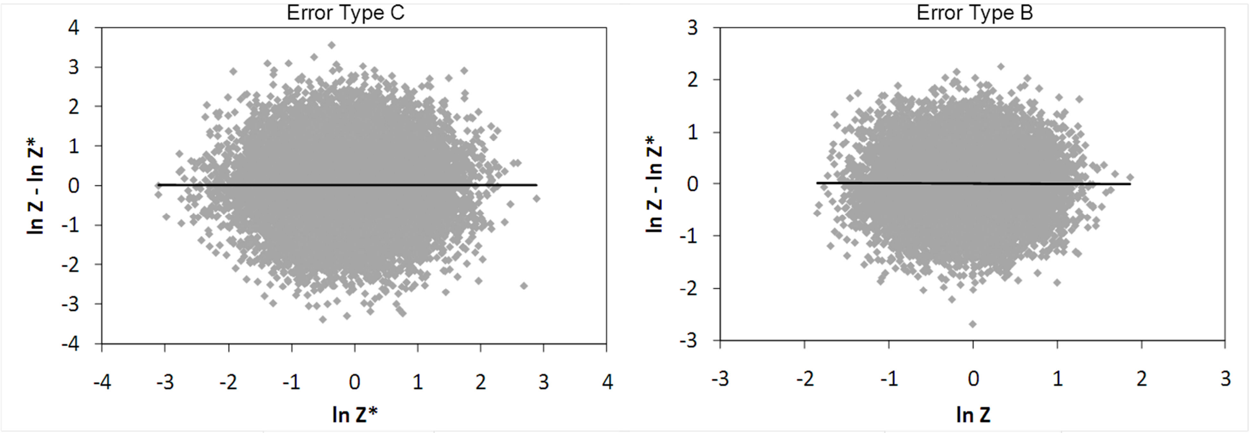

Supplement: Additional file 3 — Scatterplots of CO error ( = 0.411) versus InZ* for error type C (left panel) and versus InZ for error type B (right panel). [file 1476-069X-10-61-S3.PDF]
