# Supplementary material for: Impact of exposure measurement error in air pollution epidemiology: effect of error type in time-series studies
Source: Environ Health. 2011 Jun 22;10:61. doi: 10.1186/1476-069X-10-61 (PMC3146396; doi:10.1186/1476-069X-10-61)

Additional File 4. Figure S2. Boxplots of $R(\varepsilon_{\ln Z}, \ln Z^*)$ for 1000 simulated data time-series of error type C (top panel) and $R(\varepsilon_{\ln Z}, \ln Z)$ for 1000 simulated data time-series of error type B (bottom panel).

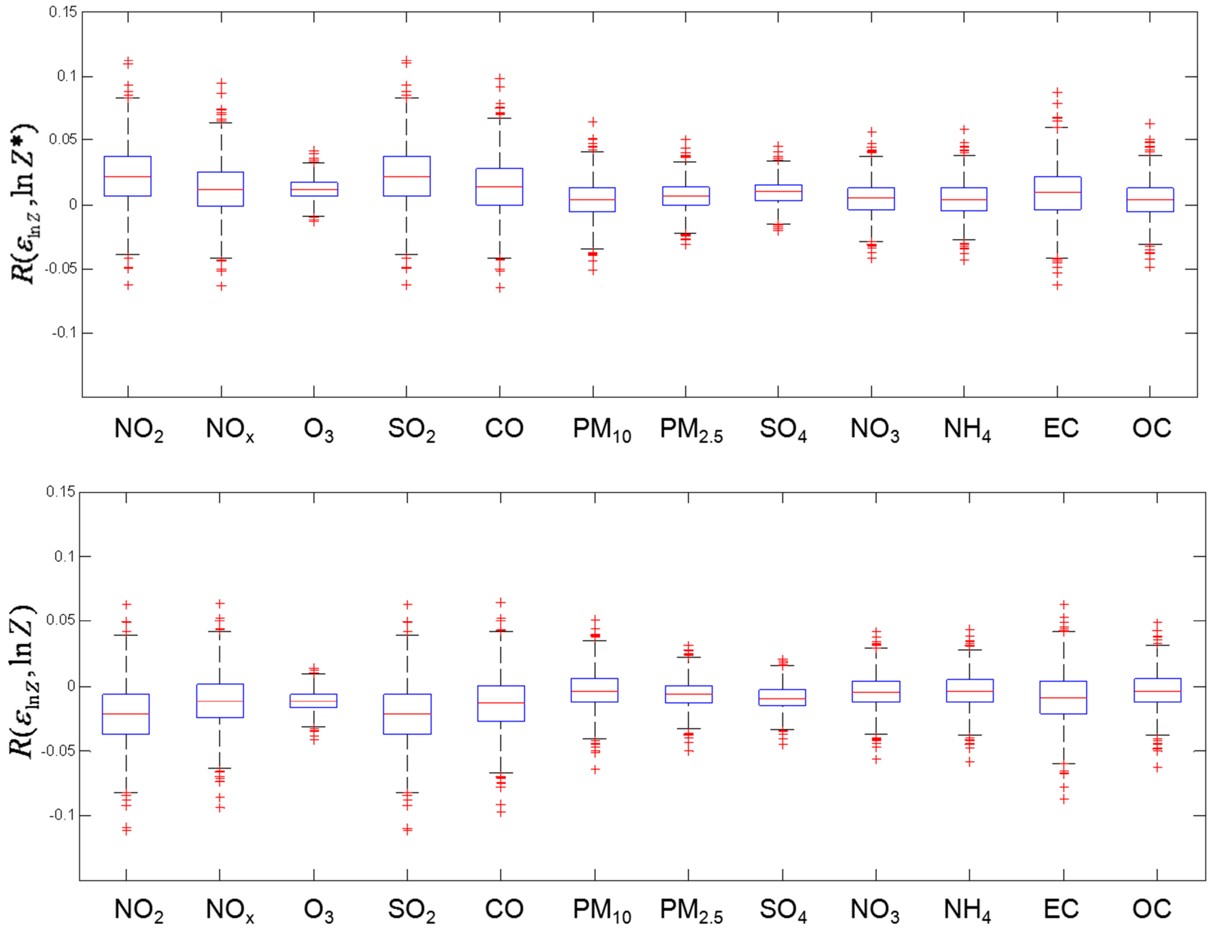

Supplement: Additional file 4 — Boxplots of R(εInZ, InZ*) for 1000 simulated data time-series of error type C (top panel) and R(εInZ, InZ) for 1000 simulated data time-series of error type B (bottom panel). [file 1476-069X-10-61-S4.PDF]
